# Visual-haptic integration with pliers and tongs: signal “weights” take account of changes in haptic sensitivity caused by different tools

**DOI:** 10.3389/fpsyg.2014.00109

**Published:** 2014-02-14

**Authors:** Chie Takahashi, Simon J. Watt

**Affiliations:** ^1^Wolfson Centre for Cognitive Neuroscience, School of Psychology, Bangor UniversityBangor, UK; ^2^Behavioural Brain Science Centre, School of Psychology, University of BirminghamBirmingham, UK

**Keywords:** tool use, multisensory integration, vision, haptic perception, cue weights

## Abstract

When we hold an object while looking at it, estimates from visual and haptic cues to size are combined in a statistically optimal fashion, whereby the “weight” given to each signal reflects their relative reliabilities. This allows object properties to be estimated more precisely than would otherwise be possible. Tools such as pliers and tongs systematically perturb the mapping between object size and the hand opening. This could complicate visual-haptic integration because it may alter the reliability of the haptic signal, thereby disrupting the determination of appropriate signal weights. To investigate this we first measured the reliability of haptic size estimates made with virtual pliers-like tools (created using a stereoscopic display and force-feedback robots) with different “gains” between hand opening and object size. Haptic reliability in tool use was straightforwardly determined by a combination of sensitivity to changes in hand opening and the effects of tool geometry. The precise pattern of sensitivity to hand opening, which violated Weber's law, meant that haptic reliability changed with tool gain. We then examined whether the visuo-motor system accounts for these reliability changes. We measured the weight given to visual and haptic stimuli when both were available, again with different tool gains, by measuring the perceived size of stimuli in which visual and haptic sizes were varied independently. The weight given to each sensory cue changed with tool gain in a manner that closely resembled the predictions of optimal sensory integration. The results are consistent with the idea that different tool geometries are modeled by the brain, allowing it to calculate not only the distal properties of objects felt with tools, but also the certainty with which those properties are known. These findings highlight the flexibility of human sensory integration and tool-use, and potentially provide an approach for optimizing the design of visual-haptic devices.

## Introduction

When humans manipulate objects with their hands while looking at them, visual and haptic size information is integrated in a manner that is highly consistent with statistically optimal models of sensory integration (Ernst and Banks, 2002; Gepshtein and Banks, 2003; Helbig and Ernst, 2007a). Such models describe how, under the assumptions that estimates from each sense are on average unbiased, and their noises are independent and Gaussian distributed, the minimum-variance unbiased estimate ($\hat{S}$_*VH*_; Equation 1) is a weighted sum of visual and haptic estimates ($\hat{S}$_*V*_, $\hat{S}$_*H*_) where the weight given to each signal (*w*_*V*_, *w*_*H*_) is proportional to the inverse of its variance (Equation 2; for a review see Oruç et al., 2003).

(1) $${\hat{S}}_{VH}=w_{V}{\hat{S}}_{V}+w_{H}{\hat{S}}_{H}$$

(2) $$w_{V}=\frac{1/\sigma_{V}^{2}}{1/\sigma_{V}^{2}+1/\sigma_{H}^{2}}\text{ where }w_{V}+w_{H}=1$$

The empirical findings that humans perform similarly to this model demonstrate that the brain ‘knows’ how much to rely on each sensory signal in a given situation. This is not trivial because relative weights of visual and haptic estimates must be adjusted moment-by-moment since they vary continuously as a function of the precise properties of particular viewing situations. For example, the reliabilities of visual and haptic size estimates almost certainly vary differently as a function of object size. And more challengingly, the reliability of visual estimates varies substantially with variations in any number of “geometrical” properties of the stimulus including the type of surface texture, the object's orientation with respect to the viewer, and viewing distance (Knill, 1998a,b; Gepshtein and Banks, 2003; Knill and Saunders, 2003; Hillis et al., 2004; Keefe et al., 2011).

Given the adeptness with which humans use tools, one might expect similar visual-haptic integration processes to operate when we manipulate objects with tools. This process is complicated, however, by the fact that in tool use haptic signals must be acquired via the handles of the tool, thereby systematically disrupting the relationship between hand opening/position and (visual) object properties. We have previously shown that, in making the decision of whether to integrate signals or not, the brain compensates for the spatial offset between visual and haptic signals introduced by simple tools (Takahashi et al., 2009). When we feel objects without a tool the degree of visual-haptic integration decreases with increasing spatial separation between signals, indicating the brain is sensitive to the probability that they refer to different objects, in which case combining them would produce errors (Gepshtein et al., 2005). We observed similar patterns of changes in visual-haptic integration in tool use, except the decision to integrate was modulated not by the separation between the hand (the origin of the haptic signal) and visual object, but by the separation between the tool tips and the object, as if the haptic signal was treated as coming from the tool-tip (Takahashi et al., 2009). This suggests the brain can correctly decide the extent to which visual and haptic information should be integrated, not based on the proximal sensory stimuli, but on their distal causes (Körding and Tenenbaum, 2006; Körding et al., 2007), taking into account the dynamics and geometry of tools.

Here we consider the problem of weighting visual and haptic “cues” (sensory estimates of size) appropriately when manipulating objects with tools. As well as spatially separating the signals, tools typically also alter the “gain” between the hand opening and object size (consider pliers and tongs, for example). In principle, this could make determining correct cue weights difficult: different tool geometries cause differences in the extent to which the haptic signal at the hand is multiplied up or down relative to object size, and the absolute sensitivity, or precision, of sensory systems generally varies with signal magnitude. Thus, different tool gains could introduce variations in the precision (reliability) of haptic size estimates that would ideally be accounted for. Here we determined the nature of the variations in the reliability of haptic size estimates with different tool geometries, and examined whether visual and haptic signals are weighted appropriately to take account of them.

There are various possibilities for how variations in tool geometry might affect the reliability of haptic size estimates, with rather different implications for what appropriate visual-haptic cue weights would be. We find it more straightforward to discuss the possible effects of different tool geometries in terms of sensitivity—Just Noticeable Differences (JNDs)—of haptic size rather than reliabilities, because experimental data and theoretical ideas such as Weber's law are typically expressed in these units. Following previous researchers (for example see Clark and Yuille, 1990; Landy et al., 1995; Knill and Richards, 1996; Ernst, 2005), we assume, however, that cue reliability relates straightforwardly to single-cue sensitivity (JND). Consider haptic size-discrimination data, measured using a standard two-interval, forced-choice (2-IFC) task, in which the participant grasps two stimuli (standard and comparison) between thumb and index finger, and reports which was larger. If the resulting data are fitted with a cumulative Gaussian psychometric function, the JND can be expressed as the standard deviation of the psychometric function which, when divided by $\sqrt{2}$, is assumed to yield the standard deviation of the underlying estimate of haptic size (σ_*H*_). The reliability of the underlying estimate is the reciprocal of its variance (1/σ^2^_*H*_).

The possible effects of variations in the gain of pliers-like tools on haptic size sensitivity could depend on either “high-level” aspects, such as how object size is ultimately represented in the brain, or “low-level” aspects, such as how the sensitivity of the basic sensory apparatus varies with hand opening. Consider first the case where the limiting factor is the precision with which different object sizes are represented in high-level processing. This could arise because the neural population that represents size contains more neurons tuned to smaller sizes and fewer tuned to larger sizes, for example, in which case absolute sensitivity to object size would decrease with increasing size. For haptic estimates of object size derived from tools to be correct we must assume that, consistent with our previous findings regarding spatial offsets (Takahashi et al., 2009), the brain is able to correctly rescale haptic signals about hand opening so that object size estimates are encoded accurately in high-level processing, independent of the tool gain. Then, if there are no significant low-level (sensory) limits then haptic sensitivity to a given object size will be determined only by the high-level constraints, and will be unaffected by the tool (i.e., the hand opening) used to hold it. Thus, in this case there would be no need to adjust cue weights to take account of tool geometry.

Although high-level limits on sensitivity must presumably exist to some degree, it is hard to envisage a system that is unaffected by altering the input signal (at the hand), and so we consider low-level factors to be more likely to limit sensitivity. Their implications are also more difficult to visualise. We therefore consider the implications of this second case in more detail. Here, we assume that underlying sensitivity to changes in hand opening is unchanged by tool use, and so the impact of different tools on haptic sensitivity to object size depends directly on (*i*) how sensitivity to hand opening varies with magnitude of hand opening, and (*ii*) the relationship between object size and the hand opening required to feel it with a given tool (the tool “gain”). In many sensory domains, the relationship between JND and stimulus magnitude is described well by Weber's law, which here implies that JNDs in hand opening should be a constant proportion of hand opening. Empirical measurements of so-called finger-span discrimination indicate, however, that while JNDs do generally increase with hand opening, they also depart significantly from Weber's law (Stevens and Stone, 1959; Durlach et al., 1989). Indeed, it can be argued that this result is unsurprising, given that judging size from hand opening requires the comparison of the positions of two “systems” (finger and thumb), each of which contains highly non-linear relationships between position and the state of muscles and joint angles (Durlach et al., 1989; Tan et al., 2007). We note, however, that, presumably due to technical challenges in presenting haptic stimuli in quick succession, previous measurements of finger-span discrimination did not use a two-interval, forced-choice task to measure sensitivity. Durlach et al. (1989), for example, used a one-interval forced-choice (is the stimulus length, *l*, or *l* + Δ*l*?). The data may therefore reflect not only perceptual sensitivity but also the precision of memory representations of size. Thus, it remains unclear whether haptic size sensitivity follows Weber's law or not.

**Figure 1:**
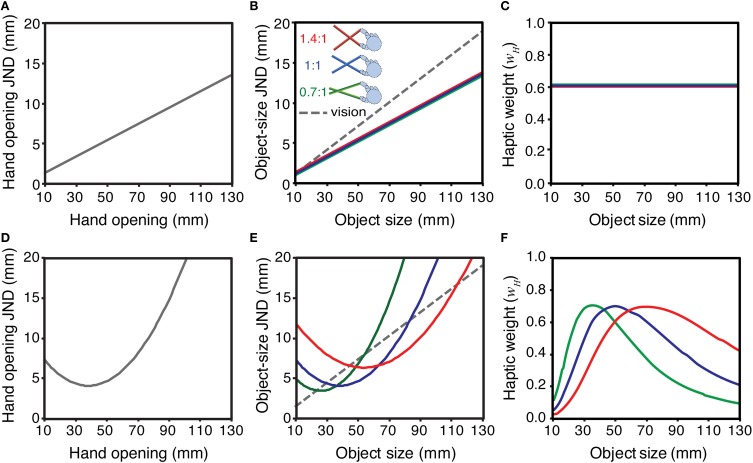
**Implications of different hypothetical hand-opening sensitivity functions for the signal weights of haptic size estimates with different tool gains**. The top row shows the case where sensitivity to hand opening (JND as a function of hand opening) follows Weber's law: **(A)** sensitivity to hand opening; **(B)** the sensitivity in A re-plotted in units of object size, with different tool gains (0.7:1, 1:1, and 1.4:1; see main text for details), and a hypothetical visual sensitivity function; **(C)** the optimal signal weights that result from the sensitivities in panel **(B)**, calculated using Equation 2. Panels **(D–F)** show the same calculations assuming a non-linear hand-opening sensitivity function.

**Figure 2:**
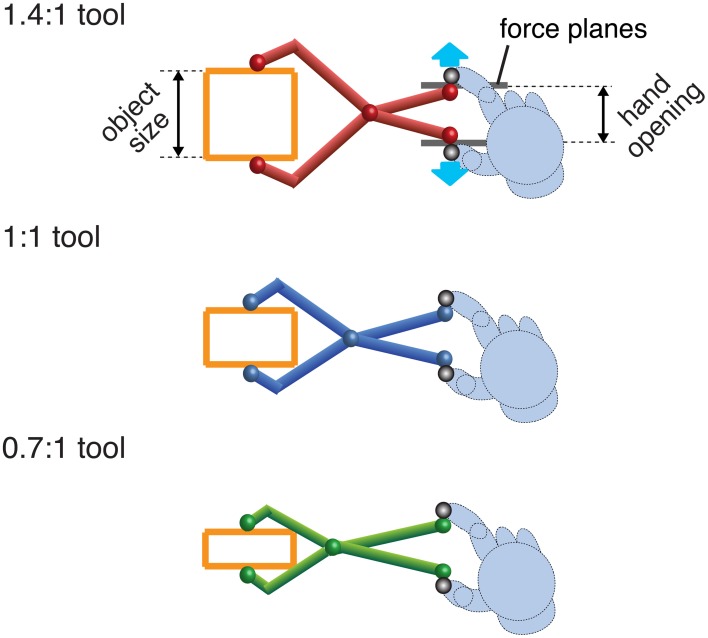
**A cartoon of the three different tools used**. Tool gain is expressed as the ratio of tool opening (object size) to hand opening. In the experiment the visual tools were rendered using 3D graphics and closely resembled these pictures. Haptic stimuli (force planes) were generated at the hand when the tool-tip touched the object. Note that the hand was not visible.

Figure 1 considers the implications of these two alternatives (Weber's law, and non-linear sensitivity functions) for weighting visual and haptic signals appropriately in tool use. The top row of panels shows the Weber's law case. Figure 1A shows a hypothetical sensitivity function for hand opening (i.e., haptic size, for an object felt directly with the hand), assuming a Weber fraction of 0.1. A similar function is also plotted for visual size, assuming a slightly different Weber fraction (0.15). Figure 1B shows sensitivity to *object size* when felt with pliers-like tools of three different gains (expressed as the ratio of tool-tip separation to hand opening; Figure 2). To calculate these values we assumed that the underlying sensitivity function for hand opening was constant, and that using the tool introduced no additional external (or internal) noise. We calculated the hand opening that would result from feeling a given object size with a particular tool (for example, feeling a 20 mm object with a 0.7:1 tool results in a hand opening of 20/0.7 = 28.6 mm). Next, we used the function in Figure 1A to “look up” the appropriate JND in *hand opening*. Finally, we transformed this “hand JND” into a JND in units of object size, by calculating the change in object size that, given the tool geometry, would produce 1 JND change at the hand. Obviously, given our assumptions, the sensitivity to changes in object size when using the 1:1 tool is the same as with no tool (Figure 1A). It can also be seen, however, that if haptic size sensitivity follows Weber's law, sensitivity to changes in object size is unaffected by tool gain. This makes intuitive sense because while the 0.7:1 tool, for example, magnifies the signal at the hand, the absolute sensitivity decreases by exactly the same amount, and so there is no net change in sensitivity to object size. Figure 1C plots the optimal cue weights for estimating object size from vision and haptics, for each of the three tool gains in Figure 1B, calculated using Equation 2. It can be seen that because both size estimates follow Weber's law, and tool gain does not affect sensitivity (or therefore reliability) of object size estimates, the *relative* reliabilities are unchanged with object size and tool gain, and so the appropriate signal weights remain constant. This is an interesting outcome in that it would simplify the brain's task, because there is no need to adjust visual and haptic weights for different tool gains. It also implies, however, that there is no opportunity to optimise haptic sensitivity in visual-haptic interfaces by using tool gain to improve haptic sensitivity.

The bottom row of panels in Figure 1 plots the same functions as the row above, but calculated assuming haptic size sensitivity at the hand is non-linearly related to hand opening (Figure 1D). The pattern is quite different to the Weber's law case. Figure 1E shows that haptic sensitivity to object size now depends directly on the tool gain and, for a given object size, can be made better or worse by choosing particular tools. Thus, if (*i*) sensitivity to changes in hand opening do not follow Weber's law, and (*ii*) low-level sensory limits directly determine haptic sensitivity to object size when using tools, the optimal visual and haptic weights for the same object in the world will change as a function of tool gain (Figure 1F), and so the brain should adjust them accordingly.

In Experiment 1 we examined how sensitivity to haptic size varies with hand opening in our experimental setup, using a two-interval, forced-choice procedure. We then measured the effect of different tool gain ratios on haptic sensitivity, using virtual tools created using a stereoscopic display and force-feedback robots. This allowed us first to establish whether or not sensitivity to object size is determined primarily by low-level sensory factors (i.e., in the manner modeled in Figure 1). Second, we could measure the shape of the haptic-size sensitivity functions with and without tools, allowing us to understand the expected effects on signal weights of different tool gains. Experiment 1 demonstrated that the reliability of haptic size estimates does vary with tool gain. We therefore examined in Experiment 2 whether the brain takes account of these changes, and adjusts signal weights based on the reliability changes induced by different tool gains. We measured weights given to the different signals at different object sizes, and with different tool gains, by measuring the perceived size of stimuli in which visual and haptic size was varied independently (so-called cue-conflict stimuli). We explore the implications of the results both for sensory integration mechanisms in visuo-motor behavior, and for the design of visual-haptic interfaces.

## Experiment 1: measurement of haptic size sensitivity

### Materials and methods

#### Participants

Six right-handed participants took part in all conditions (3 males and 3 females; 19–36 years old). All participants had normal or corrected-to-normal vision, including normal stereoacuity, and none had any known motor deficits. The participants were naïve to the purpose of the experiment. The study was approved by the School of Psychology Ethics Review Committee, Bangor University, and all participants gave informed consent before taking part.

#### Apparatus

Participants viewed 3-D stereoscopic visual stimuli in a conventional “Wheatstone” mirror stereoscope, consisting of separate TFT monitors (refresh rate 60 Hz) for each eye, centred on the body midline. Haptic stimuli were generated using two PHANToM 3.0 force-feedback robots (SenseAble Technologies, Inc.), one each for the index finger and thumb of the right hand. The robots allow participants' index fingers and thumbs to move in all six degrees of freedom (DoF), but sense and exert forces on the tips in translation (three DoF) only. The 3-D positions of the tips of the finger and thumb were continuously monitored by the robots (at 1000 Hz) and touching a virtual object resulted in appropriate reaction forces, simulating the presence of haptic surfaces in space. Participants could not see their hand, which was occluded by the stereoscope mirrors. The setup was calibrated so the visual and haptic “workspaces” were coincident. Head position was stabilized using a chin-and-forehead rest. Participants' heads were angled down approximately. 33°from straight ahead (thus, the fronto-parallel plane was angled back approximately 33°from earth-vertical).

#### Stimuli

The stimuli were positioned on a (head-centric) fronto-parallel plane, at a distance of 500 mm from the eyes. The haptic stimulus consisted of two parallel planes (stiffness = 1.05 N/mm), whose surfaces were oriented at 90° to the fronto-parallel plane. Their separation (height in the fronto-parallel plane) was varied to change object “size.”

In the no-tool condition, at the start of each trial, participants saw two spheres indicating the positions of the finger and thumb. In the tool conditions, participants saw a virtual pliers-like tool attached to the finger and thumb markers (Figure 2). Because of the 3-DoF limit on the robots' position sense and force production, and because we wanted to be sure the “opposition space” between finger and thumb was oriented orthogonally to the haptic surfaces, the visual tool was constrained to move in the fronto-parallel plane. We also presented a background fronto-parallel force plane (present in both no-tool and tool-use conditions), making it easier for participants to keep their fingers/tool in the correct orientations (a trial would not commence if the finger/thumb positions were not oriented in the fronto-parallel plane). Otherwise, the tool moved freely in this *x, y* plane, following the hand in real time, and opening and closing by rotating about the pivot (see Figure 2). Thus, the motion was akin to sliding the hand/tool along a surface such as a table, and felt intuitive and easy to carry out. We conducted pilot experiments (without a tool) to verify that the presence of the force plane did not affect size discrimination performance.

There were three differently colored tools, representing object-size: hand-opening ratios of 0.7:1 (green), and 1:1 (blue), and 1.4:1 (red) (Figure 2). Tool gain was varied by moving the position of the pivot. All tools were 18 cm long, measured from the finger position to the corresponding tool tip. Different colors were used as an aid to learning/recalling the tool geometry. When a tool-tip touched the virtual object, the appropriate force was generated at the hand.

#### Procedure

Size discrimination performance was measured in each condition using a two-interval, forced choice (2-IFC) procedure. On each stimulus interval, two visually presented “start zones” appeared (yellow spheres indicating the lateral position of the haptic stimulus, but not its size). Participants moved their hand to insert the finger/thumb spheres (no-tool condition), or the tool tips (tool condition) into the start zones, which then changed color to green, indicating that the participant should grasp the object. All visual information (including the finger/thumb spheres and visible tool) was extinguished on moving inward from the start zones, so only haptic information was available to judge object size. On each trial, participants completed two such intervals, then pushed a visual-haptic virtual button to indicate which interval contained the bigger object. Thresholds were obtained for six “base” hand openings (30, 40, 50, 60, 70, and 80 mm). Object sizes were therefore the same as the hand openings for the 1:1 tool-gain condition, and corresponded to object sizes of 21, 28, 35, 42, 49, and 56 mm in the 0.7:1 tool-gain condition, and 42, 56, 70, 84, 98, and 112 mm in the 1.4:1 tool-gain condition. On each trial the standard size was presented in one interval, and a comparison stimulus in the other, randomly ordered. A method of constant stimuli was used to generate comparison sizes, which on each trial were chosen at random from the set: base hand opening ± 1, 3, 6, or 9 mm. Base hand opening was selected at random on each trial. Participants completed 30 repetitions of each stimulus level, at all hand openings, and did not receive feedback about their performance. We did not measure performance at hand openings smaller than 30 mm because the smallest comparison stimulus (21 mm) was close to the minimum separation of the end effectors of the force-feedback robots.

To control the timing of the haptic presentation across conditions, participants were trained to grasp the stimulus for approximately 1 s in each interval and then release it. Trials on which contact time was outside the window 900–1200 ms generated an error signal, and were discarded. We added a small random jitter to the vertical position of the haptic object on each trial, so the task had to be carried out by judging object size (plane separation) rather than on the position in space of a single surface.

Trials were blocked by (*i*) no-tool, and (*ii*) tool conditions (counterbalanced order). We intermingled the three different tool-gain conditions (chosen randomly on each trial) to prevent adaptation of the relationship between felt hand opening and visual size. Participants carried out a block of practice trials in both tool and no-tool conditions to familiarise themselves with the task, and the different tools.

## Results

For each observer, in each condition, the size discrimination data were fitted with a cumulative Gaussian, using a maximum-likelihood criterion. Following previous work, JND was defined as the standard deviation (σ) of the best-fitting psychometric function (e.g., Ernst and Banks, 2002; Knill and Saunders, 2003; Hillis et al., 2004).

### Haptic sensitivity without a tool

**Figure 3:**
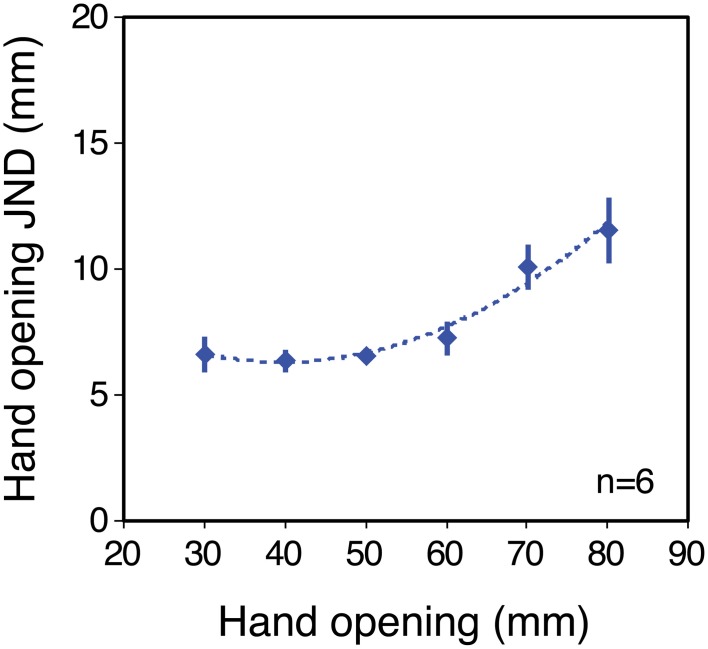
**Sensitivity to hand opening in the no-tool condition**. The figure plots JNDs in hand opening as a function of hand opening, averaged across the six observers (for individual results, see Supplementary material). The dashed line shows a second-order polynomial fit to the data, used to generate predictions for the tool conditions. Error bars denote ± 1 standard error.

Figure 3 shows size-discrimination performance (JNDs) as a function of object size, in the no-tool condition, averaged across the six participants. Clearly the average sensitivity function is non-linear, and this was reflected in the individual sensitivity functions (see Supplementary material). All participants showed increasing JNDs at large object sizes, and two out of the six showed clear non-monotonic trends, with JNDs also increasing at small sizes. For a further three JNDs appeared to have reached their minima at around 30 mm hand opening. Thus, haptic size judgements in our experiment departed substantially from Weber's law (Stevens and Stone, 1959; Durlach et al., 1989). We were unable to measure thresholds at hand openings smaller than 30 mm and so we cannot determine if all participants would have shown such increases at smaller sizes. The resting positions of the thumb and finger in natural movements correspond to non-zero hand openings (Ingram et al., 2008). Non-monotonic sensitivity could therefore in principle arise from the comparison of two position systems (for finger and thumb; Durlach et al., 1989), each of which shows decreased absolute sensitivity either side of resting position (i.e., at smaller or larger hand openings).

### Haptic sensitivity with different tool gains

**Figure 4:**
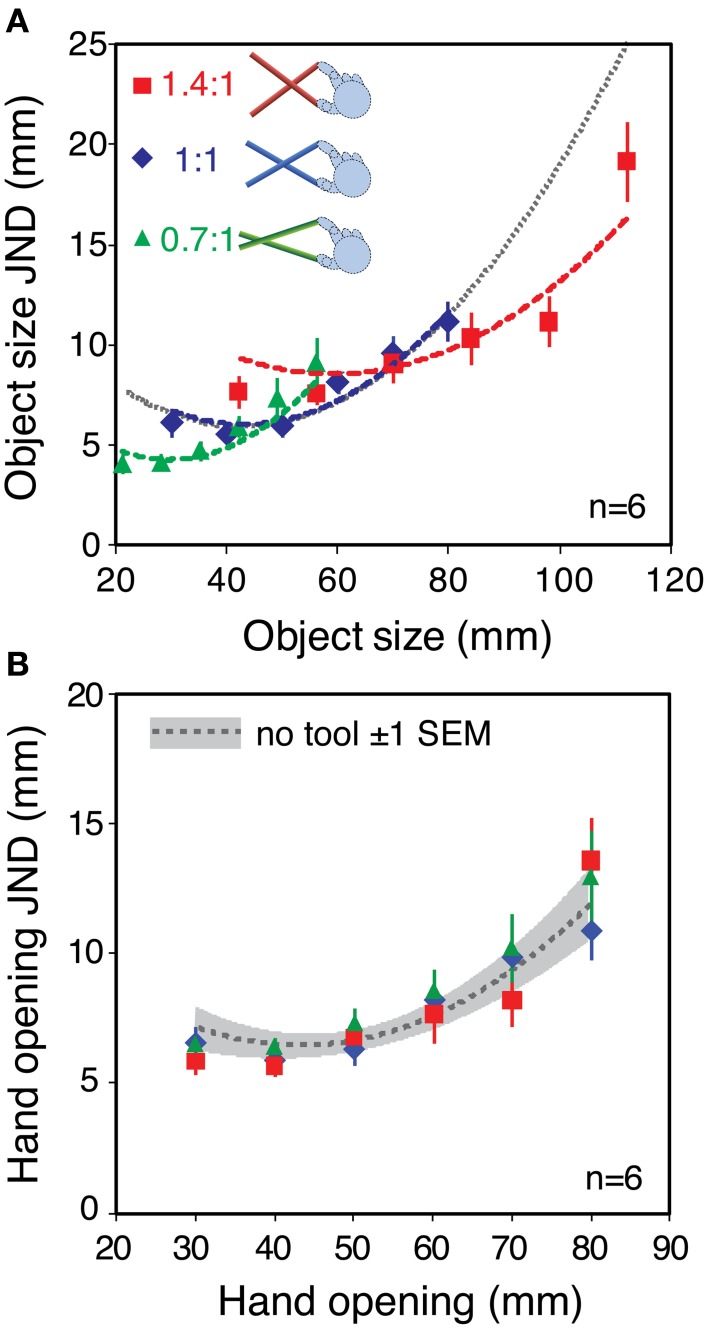
**Haptic sensitivity with different tool gains. (A)** Object size JNDs as a function of object size, averaged across the six observers. Different colors denote the different tool gains. The gray dashed line shows the fit to the no-tool data in Figure 3, extrapolated to the whole range of object sizes. This is the predicted sensitivity function is only object size *per se* determines changes in sensitivity (i.e., a high-level limit). The red, blue, and green dashed lines show the predictions assuming that low-level hand-opening sensitivity limits performance. They were calculated in the same manner as in Figure 1, by combining the fit to the basic hand-opening sensitivity with the geometrical effects of the tool (see main text). **(B)** The same data re-plotted in units of hand opening. The dashed gray line again shows the fit to the sensitivity function in the no-tool condition, and the gray zone around it shows ± 1 standard error. Error bars in both plots are standard errors.

Figure 4A plots the average sensitivity function in each of the three tool conditions. The data are plotted in “object units” (JNDs in object size as a function of object size). The gray dotted line shows the predictions for all tool-gain conditions if sensitivity is determined by high-level object representation. The curve is simply an extrapolation of the fitted polynomial function for the no-tool condition, from Figure 3. The colored dashed lines are predicted sensitivity functions for each tool condition assuming that low-level factors limit sensitivity. These are again calculated based on the polynomial fit to the average no-tool data in Figure 3, but using the calculation described in Figure 1 (i.e., assuming that size sensitivity with tools is a straightforward combination of the sensitivity to hand opening and the geometrical effects of the tool).

JNDs were very similar in the no-tool and 1:1 tool conditions, indicating that basic sensitivity was unaffected by the use of a tool *per se*. Moreover, variations in the tool gain ratio caused clear changes in sensitivity to object size. It can clearly be seen that the data are better fitted by the three separate tool curves, rather than a single sensitivity function (see Supplementary material for more details). Figure 4B re-plots the JNDs from Figure 4A in units of hand opening, rather than object-size units. If our straightforward model of sensitivity during tool use assumed in Figure 1 is correct, the sensitivities in hand-opening units should all lie on a single continuous function that represents the (unchanging) underlying sensitivity to hand opening. Figure 4B shows that this is indeed the case.

Taken together, Experiment 1 suggests that the sensitivity—and therefore in principle the reliability—of haptic size estimates sensed using tools with different gain ratios is governed primarily by perceptual sensitivity to hand opening, and not by high-level limits on size representation (see Discussion). This finding, coupled with the observed violation in Weber's law, means that the geometry of different tools does alter the reliability of haptic size estimates. A reliability-based cue-weighting process should therefore take these changes into account. We turn to this question in Experiment 2.

## Experiment 2: signal weights in tool use

Here we first established the stimulus parameters required for “two-cue” (vision-plus-haptics) conditions such that, for the same object sizes, changing tool gain should alter the reliability of the haptic signal, and so alter the signal weights. We then measured the actual weights given to each cue in these conditions using a cue-conflict paradigm.

### Methods

#### Overview

Some previous studies have used a 2-IFC task to measure performance when both visual and haptic signals are available (Ernst and Banks, 2002; Gepshtein and Banks, 2003; Gepshtein et al., 2005). This method has two key strengths. First, it allows highly accurate and precise measurement of signal weights. Participants are asked which of two intervals is larger: a cues-consistent comparison stimulus (*S*_*H*_ = *S*_*V*_), or a cue-conflict standard stimulus (*S*_*H*_ ≠ *S*_*V*_), for a range of comparison sizes. The Point of Subjective Equality (PSE) of the resulting psychometric function provides an estimate of the comparison size required to match the perceived size of the cue-conflict standard, and from this reliable measures of cue weights can be derived (Ernst and Banks, 2002). This allows quantitative tests of the observed data against point predictions. Second, it provides a measure of the discrimination threshold when both cues are available simultaneously. This is a hard test of whether information from both cues is actually integrated on individual trials because, assuming that single-cue discrimination performance represents the best the observer can achieve, improvements with two cues must indicate use of information from both sources. In contrast, a measure only of bias can resemble optimal signal weighting if the system uses a single signal on each trial, but switches between them in a reliability-dependent way (Serwe et al., 2009). Unfortunately, however, a 2-IFC task was unsuitable here because it would have required participants to compare sizes across tool conditions on a single trial. For example, to measure the perceived size of objects felt with the 1:1.4 tool the resulting percept would have to be compared with perceived size using the 1:1 tool. The tools were necessarily rendered invisible during the judgement (to control visual reliability) and this, combined with changing tool type within a trial, introduces substantial uncertainty about the tool being used on a given interval. We piloted a 2-IFC task, and found that participants were frequently confused, and therefore chose to guess on a high proportion of trials (measured discrimination performance far exceeded single-cue performance). We therefore adopted a variant of a matching task here, so as to measure perceived size (and cue weights) from trials containing a single interval. The lower accuracy and precision of this method precluded detailed quantitative evaluation of the changes in signal weights with reliability. We therefore designed the stimulus parameters for Experiment 2 to produce a qualitative change in the pattern of signal weights if reliability-based signal weighting took place. We also based our analyses on average rather than individual data.

#### Participants

Six right-handed participants took part in this experiment (2 males and 4 females; 19–36 years old). Four of them also participated in Experiment 1, but all participants were naïve to the specific purpose of this experiment. All participants had normal or corrected-to-normal vision with normal stereo acuity, and no known motor deficits. As before, the study was approved by the School of Psychology Ethics Review Committee, Bangor University, and all participants gave informed consent before taking part.

#### Apparatus and stimuli

The same apparatus was used as in Experiment 1. The haptic stimuli, and the visually defined pliers-like tools were generated in the same manner as in Experiment 1.

**Figure 5:**
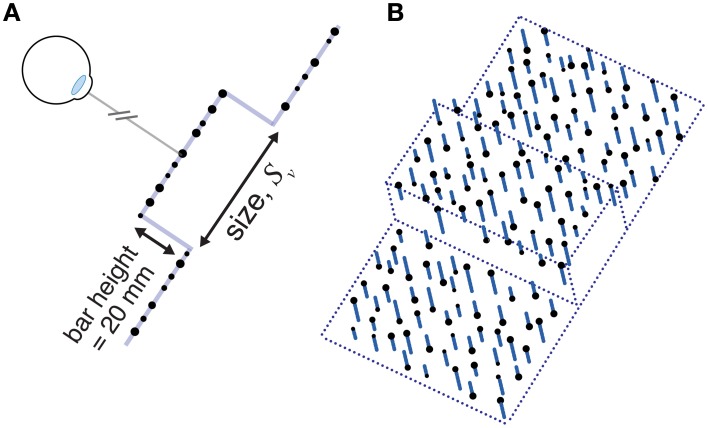
**A schematic diagram of the visual stimulus used in Experiment 2. (A)** A profile view of a stimulus with 0% noise. **(B)** A cartoon of a stimulus with non-zero visual noise. Viewing distance was 500 mm. See main text for specific details.

The visual stimulus was a rectangular object in the same position and orientation (though not necessarily the same size) as the haptic stimulus. We used a random-dot stereogram stimulus very similar to that used by Ernst and Banks (2002) so that we could vary the reliability of the visual size estimates as needed. The visual stimulus is shown schematically in Figure 5. It consisted of a random-dot-defined square “bar” represented by a plane 20 mm in front of a random-dot background plane. The whole stimulus was 80 mm wide and 200 mm tall. Visual size was the height of the bar (i.e., visual size varied in the same direction as haptic size). The dot diameter was 4.0 mm, ± up to 1.0 mm random jitter (drawn from a uniform distribution). Average dot density was 0.20 dots per mm^2^. We used anti-aliasing to achieve sub-pixel accuracy of dot positions. In addition, because random dot placement could effectively make the stimulus larger or small than intended on particular trials, we chose 3% of the dots comprising the bar, and moved them to the edges, ensuring the stimulus was always the intended size. The viewing distance to the ground plane was 500 mm. We manipulated visual reliability (added noise) in the same manner as Ernst and Banks (2002). To do this, we added a random displacement in depth to each dot, drawn from a uniform distribution, where 100% noise indicates that dot displacements were drawn from a range ±100% of the 20 mm “step” between background and bar (Figure 5).

#### Specifying the stimulus parameters

Using the same method as Experiment 1, we first measured haptic-alone size sensitivity (JNDs) for the three tool gain ratios (0.7:1, 1:1, and 1.4:1), at three object sizes: 40, 60, and 80 mm. Note that here, haptic object size (as opposed to hand opening) was the same in different tool-gain conditions, because we wanted to examine the effects on signal weights of feeling the same object with different tools. Thus, hand opening varied with tool gain. The comparison sizes in “object units” were ±1, 3, 6, and 9 mm. This meant that with the 0.7:1 tool, the standard sizes in units of hand opening were 57.1, 85.7, and 114.3 mm (comparison sizes = standard ± 1.4, 4.3, 8.6, and 12.9 mm) and with the 1.4:1 tool, standard sizes at the hand were 28.6, 42.9, and 57.1 mm (comparison = standard ± 0.7, 2.1, 4.3, and 6.4 mm).

**Figure 6:**
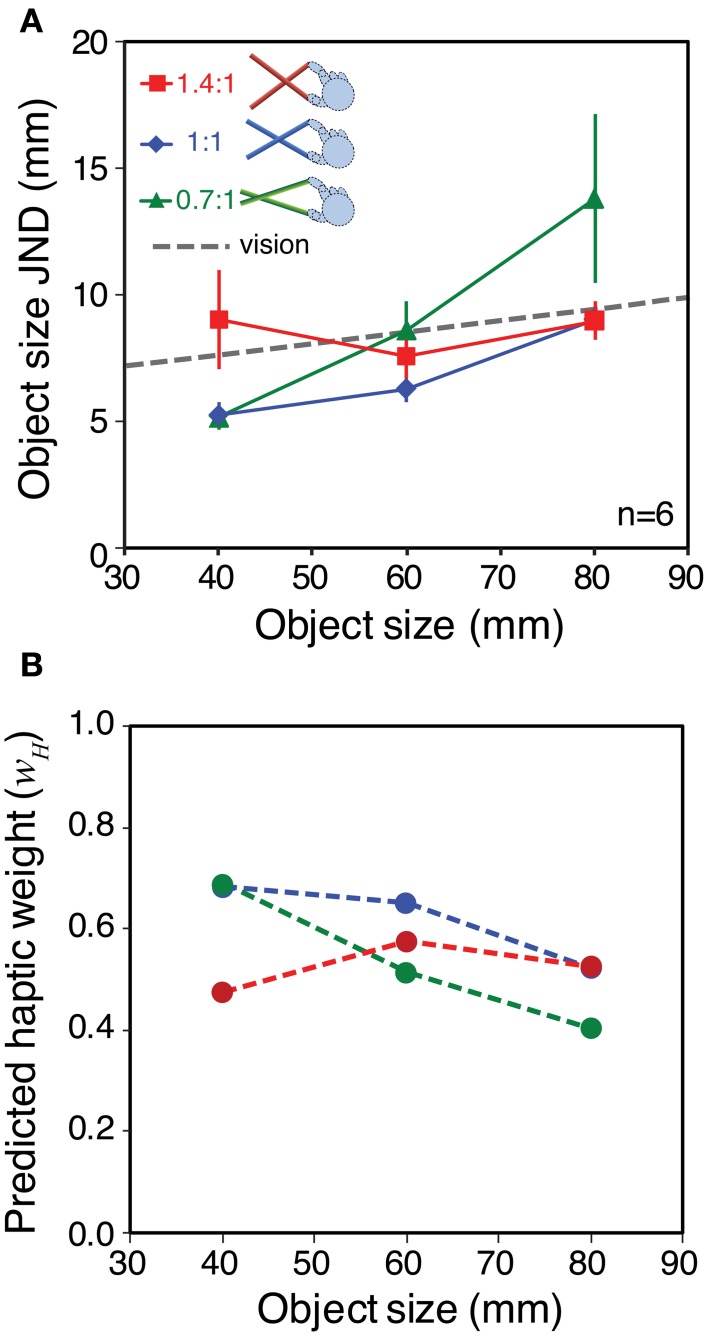
**Sensitivity to object size and predicted signal weights for Experiment 2**. **(A)** Haptic object-size JNDs for the three different object sizes, felt with each tool type. The gray dashed line shows the estimated visual sensitivity after adjusting each participant's visual noise level to approximately match his or her average haptic sensitivity (see main text). Error bars are ± 1 standard error. **(B)** Predicted haptic weights in each experimental condition, calculated using the sensitivity data from panel **(A)**, where *w*_*V*_ = 1 − *w*_*H*_

Object size sensitivity in each condition is shown Figure 6A. As we hoped, the different tool gains resulted in qualitatively different patterns of haptic sensitivity at different object sizes. Specifically, for the 40 mm object sensitivity was better with the 0.7:1 tool than with the 1.4:1 tool, and for the 80 mm object the pattern was reversed. This means we could manipulate haptic reliability in a manner that should result in clear differences in signal weights with variations in tool gain.

We wanted visual reliability not to be too low, because we wished to observe a clear contribution of both vision and haptics to the overall size estimate. Nor did we want visual reliability to be too high, because our manipulation of haptic reliability might then not have measurable effects. We therefore chose a visual noise level intended to approximately match each participant's visual sensitivity, with the 60 mm object and 1:1 tool, to his or her average haptic sensitivity across all conditions. It was not necessary to match visual reliabilities separately in all conditions because (*i*) we found in pilot testing that visual size JNDs with our stimuli varied with object size by only a small amount (0.86 mm with 20 mm variation in object size), and (*ii*) we were not testing point predictions. We therefore used data on the relationship between visual noise and JND from a previous pilot experiment (*N* = 7) as a “lookup table” to specify the visual noise levels required in each case (see Supplementary material for details). The average of the predicted visual sensitivities is shown in Figure 6A (dashed gray line).

Figure 6B shows the predicted pattern of signal weights based on the data from Figure 6A, and calculated using Equation 2 (assuming the relationship between sensitivity and reliability described in the Introduction). It can be seen that for the smallest object size, optimal integration predicts that haptics will receive more weight with the 0.7:1 tool than with the 1.4:1 tool. At the middle object size, the prediction is for similar weights (close to 0.5) for all tool types. At the largest object size (80 mm) the theory predicts a reversal of the pattern at 40 mm, with haptic weight being lower with the 0.7:1 tool, and higher with the 1.4:1 tool.

#### Procedure

We measured the perceived size of stimuli when information was available from vision and haptics simultaneously. We varied visual and haptic sizes independently (cue-conflict stimuli) to measure the weight given to each. Each trial consisted of a stimulus presentation period and a response period. In the stimulus presentation period, visual and haptic stimuli were presented simultaneously and participants explored the virtual objects using a tool. The stimulus period closely resembled the previous haptic-only trial, except for the presence of the visual stimulus. Once again the tool tips were first inserted in start zones, then all visual information (including the tool) was extinguished at the commencement of grasp closure. When the tool tips touched the haptic object, the visual stimulus appeared. As before, participants were trained to respond within a 900–1200 ms temporal window.

In the response period, a visual rectangular cuboid appeared on the screen (width 100 mm, depth 20 mm), and participants adjusted its height (in 5 mm increments from 20 to 120 mm) to match the stimulus they had just experienced. The start “size” was randomized. In pilot experiments we found similar patterns of results for visual responses and haptic responses (reporting which of a range of felt sizes matched the stimulus) and so we tested only visual responses here (Helbig and Ernst, 2007b).

In no-conflict conditions, visual object size was equal to haptic object size (40, 60, or 80 mm). In conflict conditions, visual object size was varied ± 10 mm from the haptic object size, allowing us to determine the weights given to vision and haptics (assuming *w*_*H*_ = 1 − *w*_*V*_; see below). Each participant's visual noise level was constant in all conditions, and set so as to match his or her visual and haptic sensitivities when viewing the 60 mm object and feeling it with the 1:1 tool (see earlier). The experiment was run in a series of blocks containing both no-conflict (*S*_*V*_ = *S*_*H*_) and cue-conflict (*S*_*V*_ = *S*_*H*_ ± 10 mm). Each block therefore contained 27 combinations of stimuli (3 haptic object sizes × 3 visual sizes × 3 tool gains), randomly interleaved. Each participant completed 20 judgements per stimulus combination.

## Results

**Figure 7:**
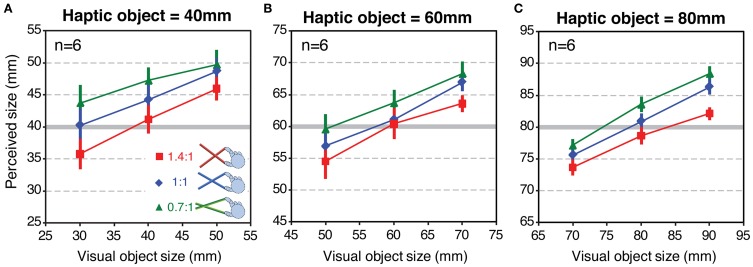
**Perceived size results from Experiment 2**. Each panel shows perceived size as a function of variations in the visual object size, for each tool type, averaged across all participants. Panels **(A–C)** show the data for haptic object sizes of 40, 60, and 80 mm, respectively. Error bars denote ± 1 standard error.

Figure 7 shows mean perceived size as a function of variation in visual object size for each tool-gain condition. The three panels show the data for the three haptic object sizes (40, 60, and 80 mm), respectively. If size estimates were based only on the haptic signal, the data would lie on horizontal lines. Conversely, if estimates relied on vision alone, the curves would lie on a line with a slope around 1.0. Clearly, the observed data are between these two extremes. This is consistent with estimates based on a weighted combination of both signals (Helbig and Ernst, 2007b). It can also be seen that, at all haptic object sizes, the data for the different tool-gain conditions are separated vertically. Relative to the 1:1 tool, perceived size was on average 2–3 mm larger with the 0.7:1 tool, which magnified the hand opening relative to object size, and a similar amount smaller with the 1.4:1 tool, which reduced the hand opening. These are relatively small effects, given the variation in actual hand opening across conditions (with the 60 mm haptic object, for example, hand openings in the three tool conditions were 85.7, 60 and 42.9 mm). This result therefore suggests that haptic size estimates when using a tool are rescaled to account for the tool gain, but that this “compensation” is incomplete (we return to this issue in the Discussion).

**Figure 8:**
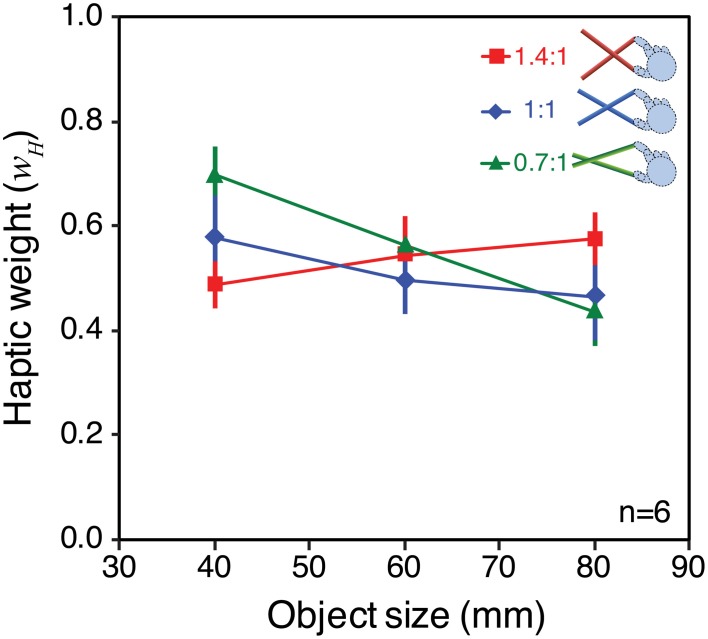
**Observed signal weights in Experiment 2**. The figure plots haptic signal weight for each “base” object size, and tool type, averaged across all participants. The weights were calculated from the effect of varying visual size in each case (the slopes of the lines in Figure 7), and assuming that *w*_*H*_ = 1 − *w*_*V*_. See main text for details of this calculation. Error bars denote ± 1 standard error.

Figure 8 plots the average weights given to vision and haptics for each combination of object size and tool gain, based on the slopes calculated from Figure 7. To calculate the weights, we assumed that perceived size ($\hat{S}$_*VH*_) is a weighted sum of visual and haptic estimates ($\hat{S}$_*V*_, $\hat{S}$_*H*_), as specified in Equations 1 and 2 (Ernst and Banks, 2002). We assumed that was unbiased. As noted above, we cannot assume that $\hat{S}$_*H*_ is unbiased. But because in each condition we fixed haptic size and varied visual size, by making the reasonable assumption that the bias in the haptic size estimate is constant for a constant object size and tool gain, the slope of the perceived size data as a function of visual size directly represents the weight given to that signal (i.e., changes in haptic bias only would shift the data up or down on the *y*-axis, but not alter the slope). The visual weight, *w*_*V*_, was therefore defined as the slope of the best fitting linear regression to the data in each case, where *w*_*H*_ = 1 − *w*_*V*_.

It can be seen that signal weights varied with both object size, and tool gain, in a manner similar to the predictions in Figure 6B. For the 40 mm object, the 0.7:1 tool resulted in more weight given to haptics, and the 1.4:1 tool resulted in more weight given to vision. As predicted, this pattern was reversed for the 80 mm object. Because we had clear predictions we did not conduct an omnibus ANOVA, but instead ran specific planned comparisons (one-tailed *t*-tests) to evaluate the statistical significance of the predicted effects. These tests showed that for the 40 mm object the weight given to haptics with 1.4:1 tool gain was significantly lower than with 0.7:1 tool gain [*t*_(5)_ = 4.92; *p* < 0.01]. For the 80 mm object, the haptic weight with the 1.4:1 tool was significantly higher than with the 0.7:1 tool [*t*_(5)_ = 2.23; *p* ≤ 0.05].

## Discussion

In Experiment 1 we found that variations in haptic size sensitivity, as measured with a 2-IFC task, did not follow Weber's law but instead followed a more complex pattern with increased hand opening. We also found that haptic size sensitivity when using virtual tools that altered the relationship between object size and hand opening was a straightforward combination of sensitivity at the hand, and the effects of the tool geometry. Thus, the “gain” of pliers-like tools alters the reliability of haptic size information, and so should be accounted for by an optimal visual-haptic integration process. In Experiment 2, we found that the brain took account of these changes in haptic reliability introduced by different tool geometries, and adjusted the weighting of haptic and visual signals in a manner broadly consistent with statistically optimal sensory integration.

Said another way, our results show that the visuo-motor system was able to adjust appropriately the weight given to size estimates from vision and haptics with changes in haptic reliability introduced by using different tool mappings. This extends our knowledge about the flexibility of sensory integration mechanisms, in particular by suggesting that the brain can represent not only distal properties of the world sensed via a “tool transformation,” but also the certainty (reliability) with which that information is known. This potentially confers the capability to combine visual and haptic signals rationally across a wide range of situations encountered in the world. Caution should be exercised in generalising our findings to the use of real tools, however. While our manipulation of tool gain accurately represented the functioning of a real tool, our virtual stimuli differed from real-world situations in several regards. In particular, movement of the tool itself was artificially constrained, and it had no perceptible mass. We used virtual stimuli to provide the degree of experimental control required for our approach, including varying visual reliability parametrically, varying visual and haptic sizes independently (to measure cue weights), and switching rapidly between tool types. But it remains to be determined whether the visuo-motor system operates similarly in real-world tool use.

### The correspondence problem in visual-haptic integration with different tool gains

On its own the finding that haptic sensitivity to object size was simply determined by the sensitivity at the hand, and the effects of tool geometry, is perhaps unsurprising: the task simply requires two signals, both of which are modified by the tool geometry in the same way, to be discriminated from one another, and the overall *magnitude* of the two estimates does not matter. That is, discrimination need not be carried out in units of the object's size in the world, taking into account the tool geometry, but could simply be carried out in the more basic units of hand opening. For integration of two different sensory signals to be effective, however (Experiment 2), the brain must transform the two signals into common units. That is, it must solve a sensory “correspondence problem”—knowing the statistics of the mapping between estimates that are sensed in fundamentally unrelated units (Ernst, 2005; Roach et al., 2006). This is important not only for establishing the relative reliabilities of signals, as studied here, but also for more fundamental aspects of sensory integration (Landy et al., 1995). For example, the combined estimate should also generate an accurate (least biased) combined estimate of the object's size, which is also not possible if the relationship between visual size and (altered) haptic size estimates is not accounted for. Moreover, knowledge of the mapping between signals is also important in making the basic decision about whether to integrate signals or not. As in other sensory domains, visual and haptic signals can often refer to different objects in the world. To avoid combining unrelated signals the brain must therefore determine how likely it is that they share a common cause (Ernst, 2005; Körding and Tenenbaum, 2006; Roach et al., 2006; Körding et al., 2007; Shams and Beierholm, 2010). Recent work suggests this process could be achieved by comparing the statistical similarity of the different signals across dimensions such as spatial location, temporal synchrony, and also signal magnitude (e.g., Deneve and Pouget, 2004; Gepshtein et al., 2005; Shams et al., 2005; Roach et al., 2006; Bresciani et al., 2006; Ernst, 2007; Knill, 2007; Körding et al., 2007; Girshick and Banks, 2009; Takahashi et al., 2009). This makes sense because the probability that two signals relate to the same object is normally directly related to how similar the estimates are.

Our method does not allow us to determine whether information was combined optimally, in the sense of producing the minimum-variance combined estimate (Ernst and Banks, 2002). Nonetheless, the observed changes in perceived size with variations in visual size in Experiment 2 are consistent with optimal integration of information from vision and haptics in all three tool-gain conditions, suggesting the brain solved this sensory correspondence problem essentially correctly. As in previous work, sensory integration occurred appropriately despite the spatial offset between visual signals (at the object) and haptic signals (at the tool handle) (Gepshtein et al., 2005; Takahashi et al., 2009). Moreover, signals appeared to be combined correctly, with appropriate weightings, across variations in tool gain. Thus, even when the proximal signals (visual size and hand opening) were discrepant visual and haptic estimates were appropriately combined on the basis of the distal object properties, taking into account the tool geometry.

#### Remapping of haptic signals

**Figure 9:**
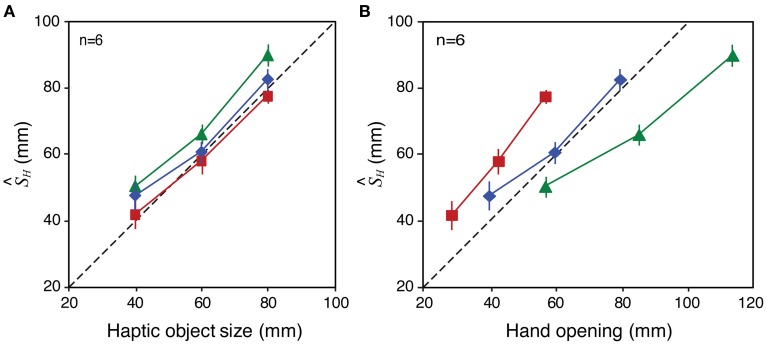
**Bias in haptic size estimates ($\hat{S}$_*H*_) with different tool gains in Experiment 2**. The figure shows $\hat{S}$_*H*_ in each condition as a function of haptic object size **(A)** and hand opening **(B)**. See main text for details of how these values were derived. Error bars denote ± 1 standard error in each case.

In principle, this correspondence of visual and haptic signals could be established by a remapping process that transforms the haptic signal at the hand to take account of the tool geometry, allowing accurate estimates of object size in the world, independent of the tool used to feel it. The perceived-size data from Experiment 2 (Figure 7) are broadly consistent with such a process. Assuming that visual size estimates were unbiased, and using our estimates of the weights given to each signal (*w*_*V*_, *w*_*H*_) we can calculate the haptic size estimate ($\hat{S}$_*H*_) by rearranging Equation 1. Figure 9 plots $\hat{S}$_*H*_ calculated in this way for each tool-gain condition as a function of haptic object size (Figure 9A) and hand opening (Figure 9B). It can be seen that perceived size estimates with gains other than 1:1 were driven predominantly by object size, and not by hand opening. Figure 9B shows for example that, for the same hand opening, haptic size estimates altered substantially as a function of tool gain. Moreover, Figure 9A shows that $\hat{S}$_*H*_ varied with haptic object size with a slope of near 1.0. This suggests the brain was transforming the proximal estimate (hand opening at the handle of the tool) in order to estimate the distal haptic size (object size in the world), allowing size estimates, and decisions about sensory integration, to be based on these common units.

An important idea in motor control is that the brain constructs internal “forward models” of limbs allowing movements of the hand and arm in space to be predicted from motor commands (Wolpert et al., 1995). In principle such a model could be extended to include representation of the tool geometry (which is relatively simple compared to the relationship between joint angles and space) allowing tools to be controlled using the same underlying systems normally used to control the arm (Takahashi et al., 2009). This is similar to the more general idea that tools are “incorporated” into our sense of body position in space—the “body schema” (Maravita and Iriki, 2004). Relatedly, there is some experimental evidence for effector-independent control processes for grasping, implying that motor outputs take tool geometry into account (Gentilucci et al., 2004; Johnson-Frey, 2004; Umilta et al., 2008; Gallivan et al., 2013).

Researchers have also studied the “reverse” of this process, showing that tool use can affect perceptual and cognitive processes including perception of the extent of peri-personal space, the allocation of spatial attention, and perception of our limbs (e.g., Farnè and Làdavas, 2000; Holmes et al., 2004; Bonifazi et al., 2007; Cardinali et al., 2009; Sposito et al., 2012), as well as motor output (Cardinali et al., 2009, 2012). These results are interesting, and point to the existence of general internal models that allow forward and reverse operations. The conclusions that can be drawn regarding the accuracy of the internal models underlying complex tool use are limited, however, for two reasons. First, although humans frequently use articulated tools, which introduce complex alterations to the hand/object mapping, the above studies focused almost exclusively on the effects of using tools that only extend reachable space. Second, the accuracy of the tool model could not be assessed because studies typically measured indirect consequences of using tools such as shifts in spatial attention, indexed by measures such as reaction time changes. Changes in motor output offer a more direct measure (e.g., Cardinali et al., 2009, 2012), but is difficult to make quantitative predictions regarding movement kinematics, particularly for aftereffects of tool use.

The finding that haptic size estimates change systematically with changes in tool gain is consistent with the existence of internal models for relatively complex transformations, allowing the magnitude of distal signals (object size) to be computed from the proximal signals (hand opening) sensed via a tool. It is interesting to note, however, that the “compensation” for tool geometry we observed, although substantial, is incomplete, $\hat{S}$_*H*_ with being biased slightly toward the actual hand opening. This could reflect uncertainty in the internal model of the tool (we do not have sufficient data to examine whether the effect reduces with time, as the internal model of the tool is refined, for example). It could also reflect uncertainty about which tool is currently being used in our task, because vision of the tool was extinguished during the size judgement to control visual reliability. Consider an estimate of the current tool gain based on a (Bayesian) combination of sensory input about what the current mapping state is, knowledge from previous experience of this mapping, and a prior for hand:object-size mapping built up from experience. Reducing uncertainty in either the sensory data or the knowledge of the mapping will lead to a greater influence of the prior (the typical mapping), which is presumably a 1:1 mapping between object size and hand opening (i.e., when there is no tool).

The full range of tool transformations that the visuo-motor system can model internally remains to be determined. In principle, equipped with an appropriate set of mathematical basis functions, any tool mapping, no matter how abstract, could be modeled. If one assumes, however, that our tool modeling capability did not evolve independently, but instead takes advantage of mechanisms that evolved for controlling our limbs in varying situations (caused by growth, fatigue, holding objects of different weights etc.), it seems likely that this architecture will impose constraints on the classes of transformation that can be modeled (consistent with findings from classical adaptation literature).

#### Estimating signal reliability in remapped haptic estimates

Neural models of population coding offer a plausible neural mechanism by which the task of appropriately weighting different sensory signals could be achieved (see for example Zemel et al., 1998; Pouget et al., 2002; Knill and Pouget, 2004; Natarajan and Zemel, 2011; Fetsch et al., 2012). These models describe how neural populations can represent the probability distribution associated with an estimate of properties of the world, and so can represent both the magnitude and uncertainty (noise) of the estimate in a manner that is analogous to statistical models of cue integration (Ernst and Banks, 2002). In simple terms, noisier inputs, caused either by internal or external factors result in “wider” population responses, and vice versa. The product of two such probabilistic distributions (one for each signal), appropriately normalized, is equivalent to the statistically optimal sensory integration described earlier (Pouget et al., 1998; Deneve et al., 2001; Ernst and Banks, 2002; Knill and Pouget, 2004).

A key feature of such a mechanism is that by operating on probability distributions it could achieve optimal, reliability-based signal weighting moment-by-moment, without requiring the explicit calculation of signal reliabilities or weights, or explicit knowledge about the circumstances under which different signals are reliable (see Natarajan and Zemel, 2011). For quantitatively meaningful outputs to emerge, however, the two neural populations for the two senses must be appropriately calibrated with respect to one another (this is the sensory correspondence problem, described above). If we assume the brain's internal tool “model” operates at the level of the whole neural population coding for haptic size, then it could effectively scale, or remap, the output of each neuron in the population according to the geometrical transformation between hand opening and object size introduced by the tool. This “single” operation would remap the whole probability distribution and so in theory would achieve both appropriate rescaling of the magnitude of the haptic size estimate and of the “width” (uncertainty) of the distribution, allowing reliability-based combination with other signals in the manner we observed.

This process also provides a mechanism by which basic sensory factors limit the reliability of high-level (object size) estimates from haptics during tool use, because the low-level noise propagates through all levels of the system. The haptic-alone discrimination performance in Experiment 1 does not, on its own, provide compelling evidence for our claim for low-level limitations on high-level haptic size estimates with tools because, as we discussed above, the task could have been carried out in hand-opening “units.” The agreement between observed and predicted signal weights in Experiment 2 suggests, however, that the single-signal results accurately reflected the system's sensitivity in the two-signal case, when haptic estimates were presumably necessarily transformed into higher level (object-size) units in order to be combined with visual size estimates. Taken together, these results suggest that haptic-size sensitivity in tool use is indeed limited by low level sensory factors and not higher-level size-representation mechanisms.

#### Rapid switching between visuo-motor mappings

The process described above—remapping between neural populations that encode the same object properties specified by different senses—could also describe “classical” adaptation, for example to prism displacement. We deliberately randomly interleaved tool types in both our experiments (on average the tool gain was 1:1) specifically to prevent such adaptation to a constant “offset.” The agreement between our predicted and observed signal weights is therefore consistent with participants switching between different visuo-motor mapping “states” on a trial-by-trial basis, and weighting signals correctly on each trial. This is consistent with other work on tool use suggesting that tool mappings are learned and can then be selected or switched by contextual information or information about tool dynamics (Imamizu et al., 2003; Massen and Prinz, 2007; Imamizu and Kawato, 2008; Botvinick et al., 2009; Beisert et al., 2010; Ingram et al., 2010). Similar ability to switch between (presumably learned) mappings has been observed in visuo-motor adaptation more generally (Cunningham and Welch, 1994). Perhaps the most commonly observed example of this is our ability to rapidly compensate for the effects of putting on and removing prescription spectacles, once we have sufficient experience with them (see Schot et al., 2012). Important questions remain, however, regarding the limitations on learning and switching between tool models, including the degree of complexity of tool transformation that can be dealt with effectively (see earlier), how many different tool models can be learned, and what are the signals that indicate the current tool mapping state to the system? Even in our relatively straightforward experiment there are several possibilities for what the system might be learning. For example, the different tools could be modeled independently, in which case information about one “tool mapping” would confer no information regarding a similar, but novel tool. Alternatively, the class of “simple gain tools” could be learned, along with a variable gain parameter, in which case our effects would transfer to novel tools of the same class. Indeed, it remains possible that nothing is learned, and that the current mapping state is recovered on each trial. Further studies are required to explore these possibilities.

#### Implications for designing tools and other visual-haptic interfaces

**Figure 10:**
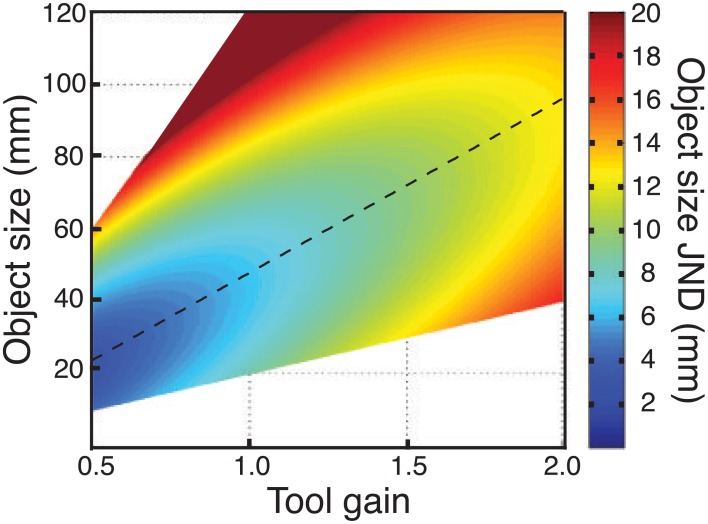
**Effects of tool gain and object size on haptic size sensitivity**. The figure plots object size JNDs as a function of both object size, and tool gains. Continuous data were obtained by using the fit to the empirical data from Experiment 1 (no-tool condition), and assuming again that sensitivity in object-size units was a straightforward combination of sensitivity to hand opening and the effects of tool geometry (Figure 1). The regions of the figure where no data are plotted correspond to hand openings beyond our measured data. JNDs ≥20 mm are not represented accurately, but are plotted as a “flat” dark red region.

Clearly there are many factors that must be borne in mind when designing tools and haptic interfaces, of which we have studied just one (haptic size sensitivity). Nonetheless, our data do provide pointers for how size sensitivity can be optimized in visual-haptic (or haptic-only) devices. The critical finding is that, because sensitivity to hand opening does not follow Weber's law, there is a particular tool gain that maximises haptic sensitivity for a particular object size. This is illustrated in Figure 10, which plots JNDs in object size as a function of both object size, and tool gain, using average data from Experiments 1 and 2, and assuming the straightforward relationship already described between sensitivity to hand opening, and sensitivity to object size with different tool gains (Experiment 1). The diagonal dashed line represents the locus of best haptic-size sensitivity in this space. In principle, armed with this information, a haptic device can be optimized for size sensitivity (similar analyses could also be carried out for other transformations). Because optimal tool gain varies continuously with object size, however, it will be critically important to answer the questions posed earlier regarding our ability to learn multiple mappings, and to switch between them, to determine how haptic interfaces are to be truly optimized for complex environments.

## Conclusions

Tools commonly change the mapping between object size and hand opening. This potentially alters the reliability of haptic size estimates, complicating the problem of weighting visual and haptic estimates correctly in sensory integration. We first confirmed that pliers-like tools do indeed introduce such changes in haptic precision, and therefore reliability. We then examined the extent to which the brain takes account of these changes in visual-haptic integration during tool use. Our results suggest that the brain compensates (albeit incompletely) for changes in proximal haptic signals introduced by different tool geometries, allowing it to dynamically and appropriately adjust the weighting given to haptic and visual signals in a manner consistent with optimal theories of sensory integration. These findings reveal high levels of flexibility of human sensory integration and tool use, as well as providing an approach for optimizing the design of visual-haptic devices.

### Conflict of interest statement

The authors declare that the research was conducted in the absence of any commercial or financial relationships that could be construed as a potential conflict of interest.

## References

[B1] Beisert M. Massen C. Prinz W. (2010). Embodied rules in tool use: a tool-switching study. J. Exp. Psychol. Hum. Percept. Perform. 36, 359–372 10.1037/a0016801 20364924

[B2] Bonifazi S. Farnè A. Rinaldesi L. Làdavas E. (2007). Dynamic size-change of peri-hand space through tool-use: spatial extension or shift of the multi-sensory area. J. Neuropsychol. 1, 101–114 10.1348/174866407X180846 19331028

[B3] Botvinick M. M. Buxbaum L. J. Bylsma L. M. Jax S. A. (2009). Toward an integrated account of object and action selection: a computational analysis and empirical findings from reaching-to-grasp and tool-use. Neuropsychologia 47, 671–683 10.1016/j.neuropsychologia.2008.11.024 19100758 PMC4126510

[B4] Bresciani J. P. Dammeier F. Ernst M. O. (2006). Vision and touch are automatically integrated for the perception of sequences of events. J. Vis. 6, 2.554–2.564 10.1167/6.5.2 16881788

[B5] Cardinali L. Frassinetti F. Brozzoli C. Urquizar C. Roy A. C. Farnè A. (2009). Tool-use induces morphological updating of the body schema. Curr. Biol. 19, R478–R479 10.1016/j.cub.2009.05.009 19549491

[B6] Cardinali L. Jacobs S. Brozzoli C. Frassinetti F. Roy A. C. Farnè A. (2012). Grab an object with a tool and change your body: tool-use-dependent changes of body representation for action. Exp. Brain. Res. 218, 259–271 10.1007/s00221-012-3028-5 22349501

[B7] Clark J. J. Yuille A. L. (1990). Data Fusion for Sensory Information Processing Systems. Boston, MA: Kluwer Academic Publishers 10.1007/978-1-4757-2076-1

[B8] Cunningham H. A. Welch R. B. (1994). Multiple concurrent visual-motor mappings: implications for models of adaptation. J. Exp. Psychol. Hum. Percept. Perform. 20, 987–999 10.1037/0096-1523.20.5.987 7964533

[B9] Deneve S. Latham P. E. Pouget A. (2001). Efficient computation and cue integration with noisy population codes. Nat. Neurosci. 4, 826–831 10.1038/90541 11477429

[B10] Deneve S. Pouget A. (2004). Bayesian multisensory integration and cross-modal spatial links. J. Physiol. Paris 98, 249–258 10.1016/j.jphysparis.2004.03.011 15477036

[B11] Durlach N. I. Delhorne L. A. Wong A. Ko W. Y. Rabinowitz W. M. Hollerbach J. (1989). Manual discrimination and identification of length by the finger-span method. Percept. Psychophys. 46, 29–38 10.3758/BF03208071 2755759

[B12] Ernst M. O. (2005). A Bayesian view on multimodal cue integration, in Human Body Perception from the Inside Out, Chapter 6, eds Knoblich G. Thornton I. M. Grosjean M. Shiffrar M. (New York, NY: Oxford University Press), 105–131

[B13] Ernst M. O. (2007). Learning to integrate arbitrary signals from vision and touch. J. Vis. 7, 7.1–7.14 10.1167/7.5.7 18217847

[B14] Ernst M. O. Banks M. S. (2002). Humans integrate visual and haptic information in a statistically optimal fashion. Nature 415, 429–433 10.1038/415429a 11807554

[B15] Farnè A. Làdavas E. (2000). Dynamic size-change of hand peripersonal space following tool use. Neuroreport 11, 1645–1649 10.1097/00001756-200006050-00010 10852217

[B16] Fetsch C. R. Pouget A. DeAngelis G. C. Angelaki D. E. (2012). Neural correlates of reliability-based cue weighting during multisensory integration. Nat. Neurosci. 15, 146–154 10.1038/nn.2983 22101645 PMC3398428

[B17] Gallivan J. P. McLean D. A. Valyear K. F. Culham J. C. (2013). Decoding the neural mechanisms of human tool use. Elife 2:e00425 10.7554/eLife.00425 23741616 PMC3667577

[B18] Gentilucci M. Roy A. C. Stefanini S. (2004). Grasping an object naturally or with a tool: are these tasks guided by a common motor representation? Exp. Brain. Res. 157, 496–506 10.1007/s00221-004-1863-8 15007584

[B19] Gepshtein S. Banks M. S. (2003). Viewing geometry determines how vision and haptics combine in size perception. Curr. Biol. 13, 483–488 10.1016/S0960-9822(03)00133-7 12646130

[B20] Gepshtein S. Burge J. Ernst M. O. Banks M. S. (2005). The combination of vision and touch depends on spatial proximity. J. Vis. 5, 1013–1023 10.1167/5.11.7 16441199 PMC2632311

[B21] Girshick A. R. Banks M. O. (2009). Probabilistic combination of slant information: weighted averaging and robustness as optimal percepts. J. Vis. 9, 8.1–8.20 10.1167/9.9.8 19761341 PMC2940417

[B22] Helbig H. B. Ernst M. O. (2007a). Optimal integration of shape information from vision and touch. Exp. Brain Res. 179, 595–606 10.1007/s00221-006-0814-y 17225091

[B23] Helbig H. B. Ernst M. O. (2007b). Knowledge about a common source can promote visual-haptic integration. Perception 36, 1523–1533 10.1068/p5851 18265835

[B24] Hillis J. M. Watt S. J. Landy M. S. Banks M. S. (2004). Slant from texture and disparity cues: optimal cue combination. J. Vis. 4, 967–992 10.1167/4.12.1 15669906

[B25] Holmes N. P. Calvert G. A. Spence C. (2004). Extending or projecting peripersonal space with tools? Multisensory interactions highlight only the distal and proximal ends of tools. Neurosci. Lett. 372, 62–67 10.1016/j.neulet.2004.09.024 15531089

[B26] Imamizu H. Kawato M. (2008). Neural correlates of predictive and postdictive switching mechanisms for internal models. J. Neurosci. 28, 10751–10765 10.1523/JNEUROSCI.1106-08.2008 18923050 PMC6671335

[B27] Imamizu H. Kuroda T. Miyauchi S. Yoshioka T. Kawato M. (2003). Modular organization of internal models of tools in the human cerebellum. Proc. Natl. Acad. Sci. U.S.A. 100, 5461–5466 10.1073/pnas.0835746100 12704240 PMC154367

[B28] Ingram J. N. Howard I. S. Flanagan J. R. Wolpert D. M. (2010). Multiple grasp-specific representations of tool dynamics mediate skilful manipulation. Curr. Biol. 20, 618–623 10.1016/j.cub.2010.01.054 20346672 PMC3501566

[B29] Ingram J. N. Körding K. P. Howard I. S. Wolpert D. M. (2008). The statistics of natural hand movements. Exp. Brain Res. 188, 223–236 10.1007/s00221-008-1355-3 18369608 PMC2636901

[B30] Johnson-Frey S. H. (2004). The neural bases of complex tool use in humans. Trends Cogn. Sci. 8, 71–78 10.1016/j.tics.2003.12.002 15588811

[B31] Keefe B. D. Hibbard P. B. Watt S. J. (2011). Depth-cue integration in grasp programming: no evidence for a binocular specialism. Neuropsychologia 49, 1246–1257 10.1016/j.neuropsychologia.2011.02.047 21371484

[B32] Knill D. C. (1998a). Discrimination of planar surface slant from texture: human and ideal observers compared. Vis. Res. 38, 1683–1711 10.1016/S0042-6989(97)00325-8 9747503

[B33] Knill D. C. (1998b). Ideal observer perturbation analysis reveals human strategies for inferring surface orientation from texture. Vis. Res. 38, 2635–2656 10.1016/S0042-6989(97)00415-X 12116709

[B34] Knill D. C. (2007). Robust cue integration: a bayesian model and evidence from cue-conflict studies with stereoscopic and figure cues to slant. J. Vis. 7, 5.1–5.24 10.1167/7.7.5 17685801

[B35] Knill D. C. Pouget A. (2004). The Bayesian brain: the role of uncertainty in neural coding and computation. Trends Neurosci. 27, 712–719 10.1016/j.tins.2004.10.007 15541511

[B36] Knill D. C. Richards W. (eds.). (1996). Perception as Bayesian Inference. New York, NY: Cambridge University Press

[B37] Knill D. C. Saunders J. A. (2003). Do humans optimally integrate stereo and texture information for judgments of surface slant? Vis. Res. 43, 2539–2558 10.1016/S0042-6989(03)00458-9 13129541

[B38] Körding K. P. Beierholm U. Ma W. J. Quartz S. Tenenbaum J. B. Shams L. (2007). Causal inference in multisensory perception. PLoS ONE 9:e943 10.1371/journal.pone.0000943 17895984 PMC1978520

[B39] Körding K. P. Tenenbaum J. B. (2006). Causal inference in sensorimotor integration, in Advances in Neural Information Processing Systems 19 (NIPS). eds Schölkopf B. Platt J. Hofmann T. (Cambridge, MA: MIT Press), 737–744

[B40] Landy M. S. Maloney L. T. Johnston E. B. Young M. (1995). Measurement and modelling of depth cue combination: in defense of weak fusion. Vis. Res. 35, 389–412 10.1016/0042-6989(94)00176-M 7892735

[B41] Maravita A. Iriki A. (2004). Tools for the body (schema). Trends Cogn. Sci. 8, 79–86 10.1016/j.tics.2003.12.008 15588812

[B42] Massen C. Prinz W. (2007). Programming tool-use actions. J. Exp. Psychol. Hum. Percept. Perform. 33, 692–704 10.1037/0096-1523.33.3.692 17563230

[B43] Natarajan R. Zemel R. S. (2011). Dynamic cue combination in distributional population code networks, in Sensory Cue Integration, Chapter 5, eds Tromershäuser J. Körding K. Landy M. S. (New York, NY: Oxford University Press), 368–392

[B44] Oruç Ý. Maloney L. T. Landy M. S. (2003). Weighted linear cue combination with possibly correlated error. Vis. Res. 43, 2451–2468 10.1016/S0042-6989(03)00435-8 12972395

[B45] Pouget A. Deneve S. Duhamel J. (2002). A computational perspective on the neural basis of multisensory spatial representations. Nat. Rev. Neurosci. 3, 741–747 10.1038/nrn914 12209122

[B46] Pouget A. Zhang K. Deneve S. Latham P. E. (1998). Statistically efficient estimation using population codes. Neural Comput. 10, 373–401 10.1162/089976698300017809 9472487

[B47] Roach N. W. Heron J. McGraw P. V. (2006). Resolving multisensory conflict: a strategy for balancing the costs and benefits of audio-visual integration. Proc. R. Soc. B 273, 2159–2168 10.1098/rspb.2006.3578 16901835 PMC1635528

[B48] Schot W. Brenner E. Sousa R. Smeets J. B. (2012). Are people adapted to their own glasses? Perception 41, 991–993 10.1068/p7261 23362676

[B49] Serwe S. Drewing K. Trommershäuser J. (2009). Combination of noisy directional visual and proprioceptive information during visually-guided pointing. J. Vis. 9, 28.1–28.14 10.1167/9.5.28 19757906

[B50] Shams L. Beierholm U. R. (2010). Causal inference in perception. Trends Cogn. Sci. 14, 425–432 10.1016/j.tics.2010.07.001 20705502

[B51] Shams L. Ma W. J. Beierholm U. (2005). Sound-induced flash illusion as an optimal percept. Neuroreport 16, 1923–1927 10.1097/01.wnr.0000187634.68504.bb 16272880

[B52] Sposito A. Bolognini N. Vallar G. Maravita A. (2012). Extension of perceived arm length following tool-use: clues to plasticity of body metrics. Neuropsychologia 50, 2187–2194 10.1016/j.neuropsychologia.2012.05.022 22683448

[B53] Stevens S. S. Stone G. (1959). Finger span: ratio scale, category scale, and JND scale. J. Exp. Psychol. 57, 91–95 10.1037/h0048829 13641579

[B54] Takahashi C. Diedrichsen J. Watt S. J. (2009). Integration of vision and haptics during tool use. J. Vis. 9, 3.1–3.13 10.1167/9.6.3 19761294

[B55] Tan H. Z. Srinivasan M. A. Reed C. M. Durlach N. I. (2007). Discrimination and identification of finger joint-angle positions using active motion. ACM Trans. Appl. Percept. 4 10.1–10.14 10.1145/1265957.1265959

[B56] Umilta M. A. Escola L. Instkirvell I. Grammont F. Rochat M. Caruana F. (2008). When pliers become fingers in the monkey motor system. Proc. Natl. Acad. Sci. U.S.A. 105, 2209–2213 10.1073/pnas.0705985105 18238904 PMC2538900

[B57] Wolpert D. M. Ghahramani Z. Jordan M. I. (1995). An internal model for sensorimotor integration. Science 269, 1880–1882 10.1126/science.7569931 7569931

[B58] Zemel R. S. Dayan P. Pouget A. (1998). Probabilistic interpretation of population codes. Neural Comput. 10, 403–430 10.1162/089976698300017818 9472488

