# Supplementary material for: Visual-haptic integration with pliers and tongs: signal “weights” take account of changes in haptic sensitivity caused by different tools
Source: Front Psychol. 2014 Feb 14;5:109. doi: 10.3389/fpsyg.2014.00109 (PMC3924038; doi:10.3389/fpsyg.2014.00109)
Supplement: Supplementary file 1 [file DataSheet1.PDF]

SUPPLEMENTARY MATERIAL

# Visual-haptic integration with pliers and tongs: signal 'weights' take account of changes in haptic sensitivity caused by different tools

Chie Takahashi and Simon Watt

## 1. Individual hand-opening JNDs in Expt. 1.

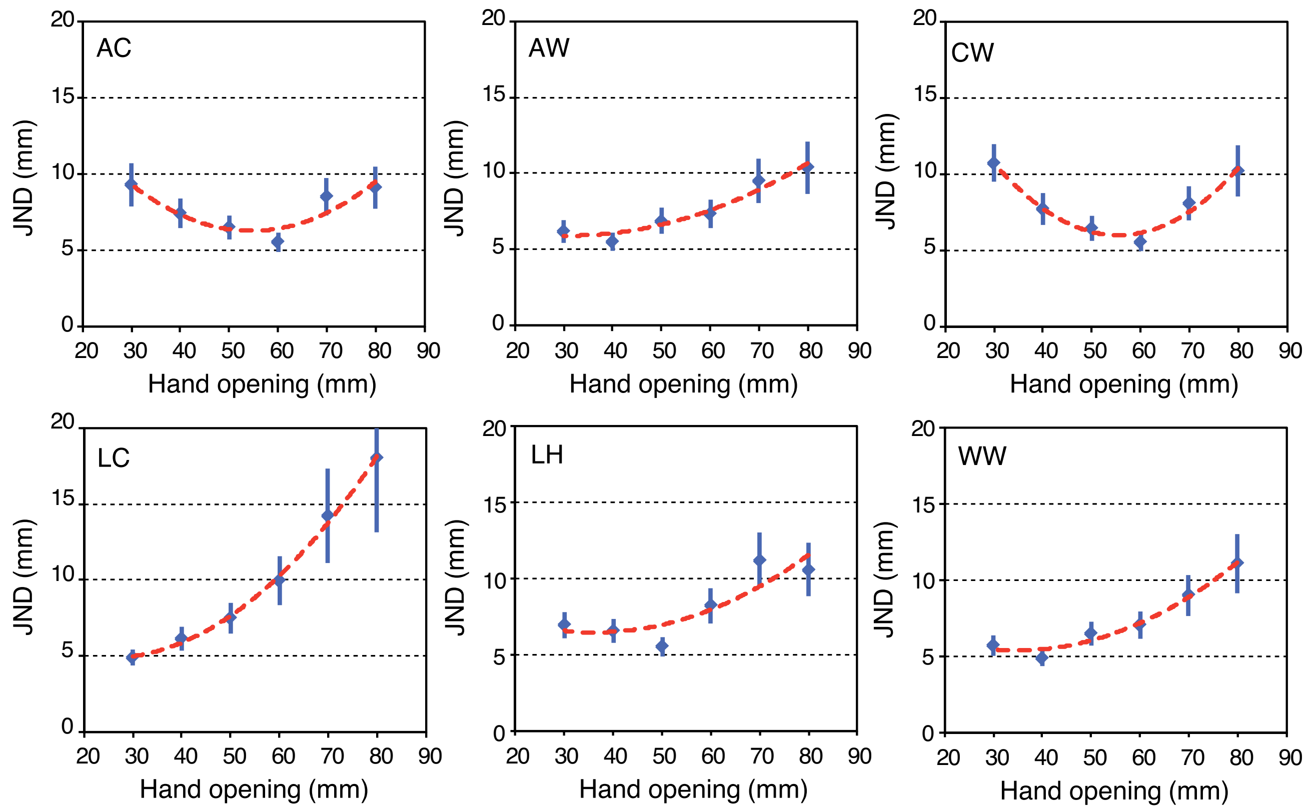


**Figure S1.** Individual participant's sensitivity to hand opening in the no-tool condition. JNDs in hand opening are plotted as a function of hand opening. The dashed line shows a second-order polynomial fit to the data. Error bars denote ±1 standard error.

## 2. Fit of data to predictions in Expt. 1.

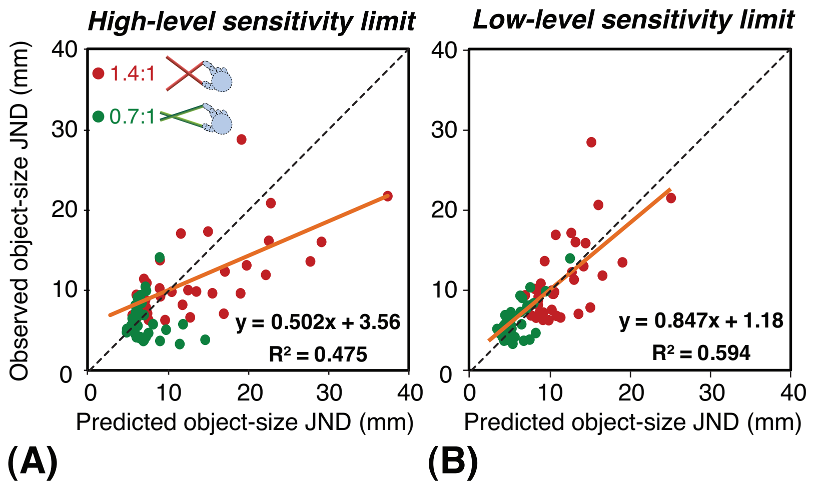


**Figure S2.** Scatterplot of the correlation between observed and predicted object-size JNDs in Expt. 1, for non-1:1 tool gains. (A) Thresholds determined by high-level size representation (constant object-size JNDs with different tool gains), and (B) by low-level sensitivity to hand opening, combined with the geometric effect of tool gain. Linear regression parameters (including $R^2$) are shown in each plot.

[1] Wolfson Centre for Cognitive Neuroscience, School of Psychology, Bangor University, Wales, UK
[2] Behavioural Brain Science Centre, School of Psychology, University of Birmingham, UK

## 3. Specifying the visual stimulus parameters

Our goal was to estimate a visual noise value for each participant that would approximately match visual sensitivity with their average haptic sensitivity, across the various conditions.

For a previous experiment we characterised the relationship between visual noise in our stimulus and JNDs in visual size. This was done using a very similar 2-IFC method to the haptic size JND measurements described in the main text, and using the same analysis techniques. Here, we used two standard sizes, 50 and 80 mm. Noise values were different for each participant. The comparison sizes were equal to the standard size ±1, 3, 6, & 9 mm. A fixation cross first briefly appeared indicating the position of the upcoming stimulus (but not its size), and the two stimulus intervals were then presented for 1 sec each, separated by a 1.6 sec inter-stimulus interval. This time was determined based on the typical inter-stimulus interval required for haptic trials. Observers indicated which interval contained the larger size.

Figure S3 shows the JNDs from seven participants. In each case the data are well fitted by a second-order polynomial fit. It can be seen that the effect of noise on JNDs is broadly similar for all the participants, and so we used the average of these data to specify parameters for the present experiment.

Because we needed to estimate visual sensitivity for an intermediate size (60 mm) we checked that JNDs scaled linearly with size, by measuring JNDs in several of the current participants, using the method above, at four object sizes (30, 50, 70, & 90 mm), and two noise levels (150 and 300%). Figure S4A shows an example observer. Because the data were well fit by a linear function, we could estimate the noise level required to produce a target visual JND simply by interpolating linearly between the average 'noise functions' for 50 and 80 mm objects (Figure S4B), generated by averaging the individual fits in Figure S3.

While this procedure could appear convoluted, note that the alternative approach, of mapping out JNDs for each participant as a function of both size and noise level, would have required approximately 16-20 hours of testing per participant, and was therefore not practicable.

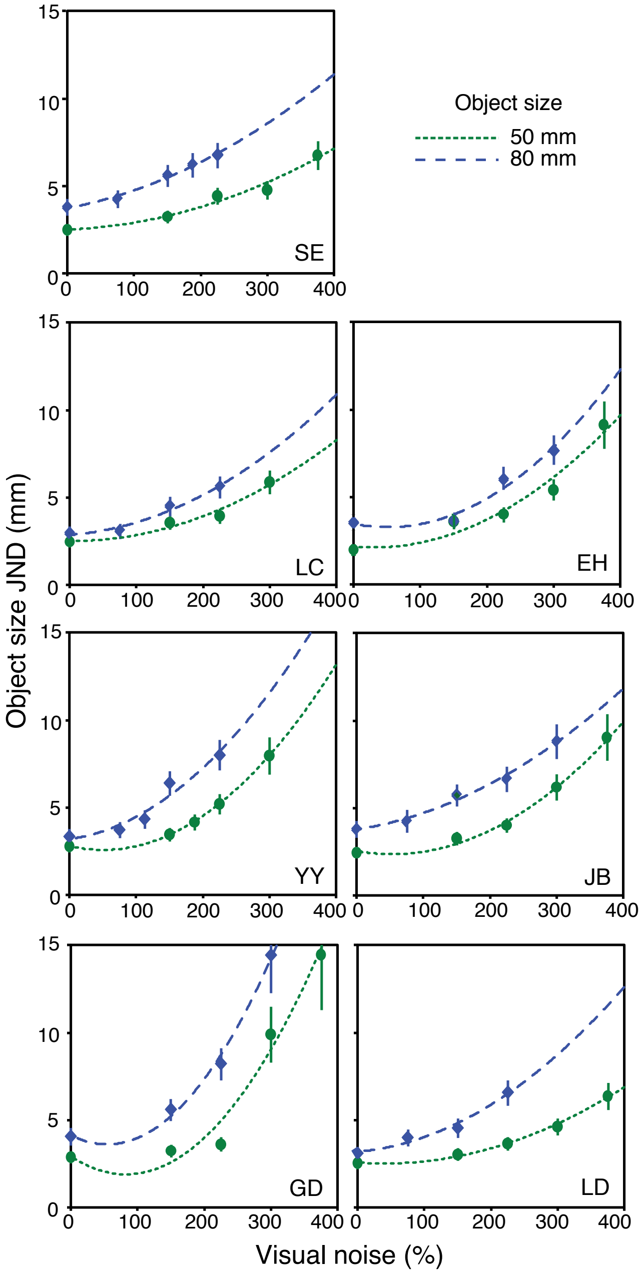


**Figure S3.** Individual object size JND data as a function of visual noise, from a previous pilot experiment in our lab. Error bars denote ±1 SEM.

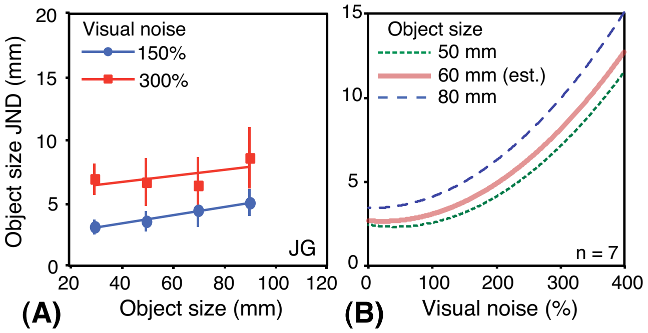


Figure S4. (A) Example data for the effect of object size on JNDs. Error bars denote ±1 SEM. (B) Average effects of visual noise on JNDs from Figure S3, and estimate for 60 mm (obtained by linear interpolation).
